# Avascular Necrosis of the Tibial Plafond and Talus After Intra-articular Corticosteroid Injection: A Case Report

**DOI:** 10.7759/cureus.81245

**Published:** 2025-03-26

**Authors:** Izabela A Pop, Steven D Steinlauf, Marissa C Tandron

**Affiliations:** 1 Medicine, University of Miami Miller School of Medicine, Miami, USA; 2 Orthopedic Surgery, University of Miami Miller School of Medicine, Miami, USA

**Keywords:** avascular necrosis, avascular osteonecrosis, steroid injection complication, talus avn, tibial plafond

## Abstract

A 61-year-old female presented 13 months after a fall down three stairs on September 14, 2021. Her chief complaint consisted of anterior and lateral ankle pain. Initial imaging studies, including an MRI in February 2022, were unremarkable. She underwent conservative treatment for soft tissue impingement syndrome under the care of a podiatric surgeon. This included two corticosteroid injections. After the second injection, she reported an increase in pain. She did not sustain any new trauma. An MRI that was taken in April of 2022, two months after her second steroid injection, demonstrated avascular necrosis (AVN) of the talus and tibial plafond. In an effort to decrease her symptoms associated with impingement syndrome, arthroscopic debridement focusing on the removal of scar tissue and synovitis was performed. It was decided to observe the areas of avascular bone, secondary to inherent risks in the surgical management of AVN. In addition, it was unknown how much of the pain was coming from the avascular process versus the impingement syndrome. Her symptoms have improved, but she continues to have mild ankle pain and limitations. Although a rare complication, this case demonstrates the risk of developing ankle AVN after receiving a corticosteroid injection. When confronted with ongoing ankle pain, advanced imaging may be warranted to rule out AVN.

## Introduction

Avascular necrosis (AVN) is a condition caused by impaired perfusion of a bone that leads to ischemia and eventually tissue death. It can be incited by a traumatic event that injures the blood supply or idiopathic events. Long-term corticosteroid use is a known cause of AVN. In vitro studies have shown that corticosteroid injections can lead to chondrocyte toxicity and a decrease in viability [1]. Our colleagues in the hip and knee joint reconstruction specialty have conducted studies to determine if intra-articular corticosteroid injections can cause AVN or lead to its progression. While their data on long-term effects have been conflicting, the rate of new AVN or progression of AVN after injections in short-term follow-up is around 2% to 17% in studies [2-4]. It is estimated that the incidence of osteonecrosis in the United States is 20,000-30,000, and it is the cause of 10% of the 250,000 hip replacements that happen annually [5]. AVN of the talus is much less common than in the femoral head and is typically caused by trauma, although other causes, including steroid use, alcohol use, and systemic causes like sickle cell disease, need to be excluded [1]. Presentation of AVN varies, but it can be painful or asymptomatic. We report a case of AVN of the talus and tibial plafond seven months after a low-energy fall that resulted in no fractures. The only potential risk factor that could be identified was an intra-articular corticosteroid injection. This case illustrates the potential for AVN of the talus and tibia after a common therapeutic modality, utilized for many intra-articular pathologies.

## Case presentation

A 61-year-old female with a history of a fall down three stairs on September 14, 2021, presented to our institution in October of 2022 with pain and swelling over the anterior ankle and laterally in the region of the fibula. She did not complain of instability. Her initial care by a podiatrist at an outside facility began in September of 2021 and included a Medrol Dosepak, a six-day taper of 21 tablets of 4 mg methylprednisolone over six days, initially after the trauma. For the first three months after her fall, she utilized a cam walker boot, which had no impact on her pain. She then performed physical therapy, which made the pain worse.

Initial radiographs from September 2021 were reportedly “negative,” and an MRI from February 8, 2022, did not demonstrate any bone marrow edema, fracture, or AVN.

Her treating podiatrist performed two corticosteroid injections. The first five months after her initial trauma and after her first ankle MRI gave her temporary relief. The second shot, six months after her initial trauma, resulted in a marked increase in her pain. A repeat MRI scan from April 15, 2022, seven months after the trauma, demonstrated AVN of the posterior talar body and tibial plafond as well as a fracture of the talar body. Therefore, the patient was referred to us for treatment. She reported constant pain, moderate swelling, night pain, and no instability. Her symptoms were worse with walking and standing and better at rest.

She has a past medical history of hypertension, hyperlipidemia, and atrial fibrillation. Social history revealed only one alcoholic drink per week. No risk factors for AVN were identified. Physical exam demonstrated a height of 155 cm, 89.4 kg, and a calculated body mass index of 37.3 kg/m². On foot and ankle examination, the dorsalis pedis pulse was not palpable, and the posterior tibial pulse was 2+. Her ankle was moderately swollen. There was full subtalar and midfoot range of motion without pain. Ankle range of motion was dorsiflexion of 5 degrees on the left and 15 degrees on the right side, and plantarflexion was 45 degrees on the left with pain and 55 degrees without pain on the right. She had a significant anterior drawer, but it was symmetrical bilaterally at 1-2+. Minimal talar tilt with guarding was noted. Strength was 5/5, and sensation was intact.

**Figure 1:**
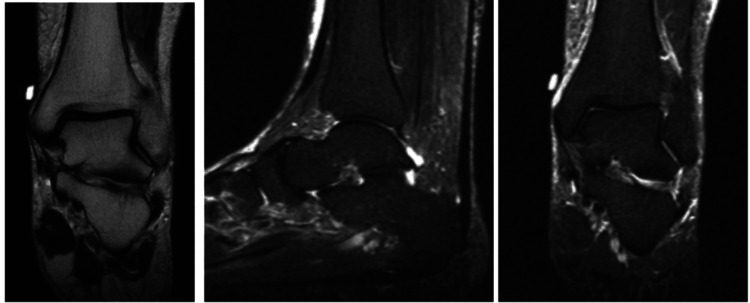
Initial MRI from February 8, 2022. No bone marrow edema or AVN and no fractures. AVN: avascular necrosis

**Figure 2:**
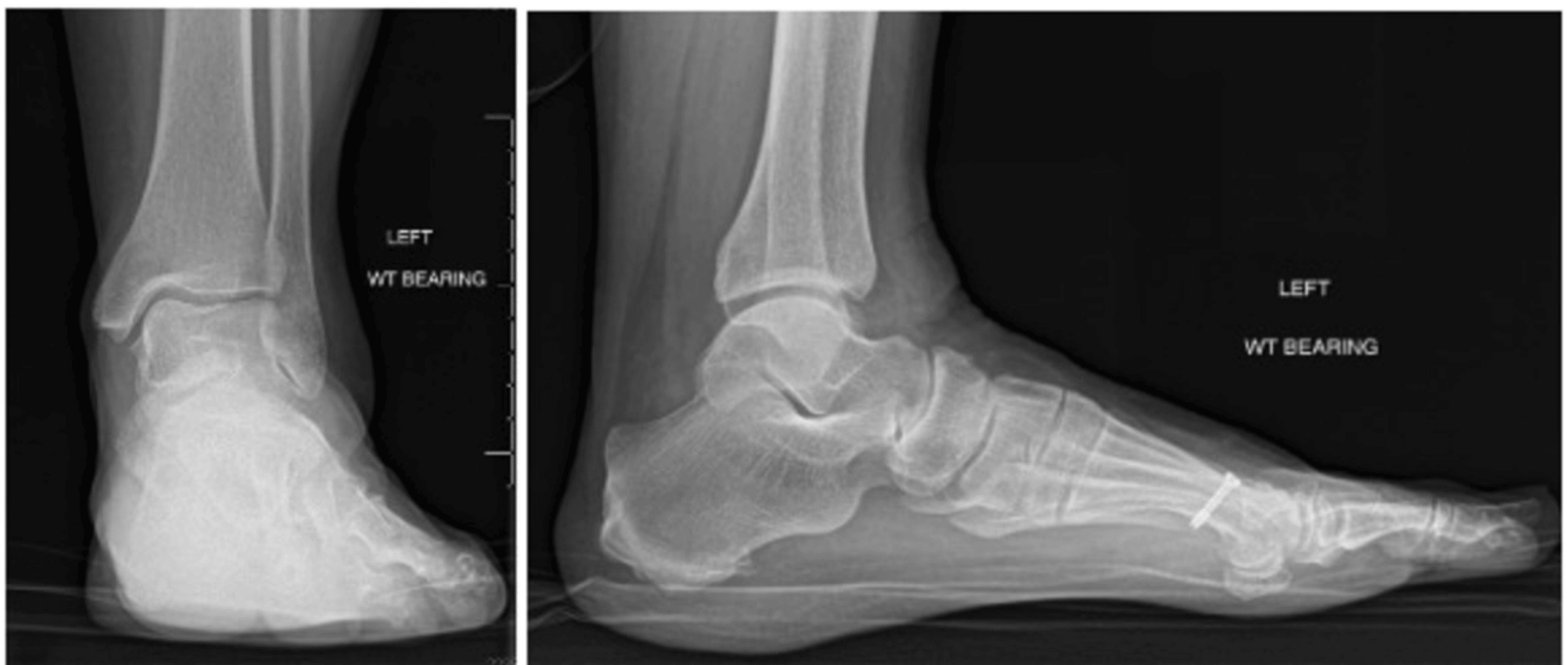
WB X-ray 13 months post-inciting event and approximately seven months after the development of AVN. WB: weight bearing; AVN: avascular necrosis

**Figure 3:**
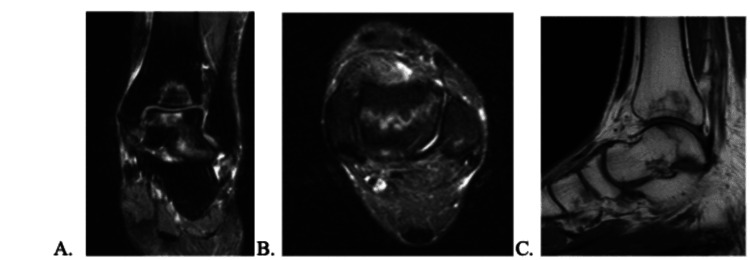
MRI that initially showed AVN on April 15, 2022. In addition, there is a nondisplaced fracture of the talar body. AVN: avascular necrosis

**Figure 4:**
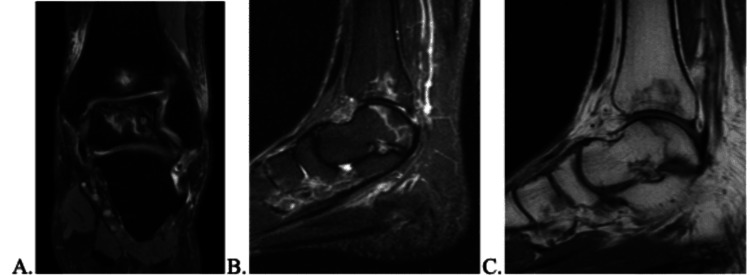
MRI from September 14, 2022, with no progression but continuation of AVN. AVN: avascular necrosis

Upon presentation to our office, radiography of the left ankle with weight-bearing views from October 7, 2022, showed no evidence of osteochondral lesion or fracture. No articular collapse was noted (Figure 1). Multiple MRIs were reviewed as noted above. The first MRI was an MRI of the left ankle from October 19, 2021. There was no obvious osseous pathology in the talar dome or the tibial plafond by report. The second MRI, performed five months after the fall and before the second corticosteroid shot, shows no osteochondral pathology or AVN (Figure 2). On an MRI seven months after the initial trauma and one and a half months after the second corticosteroid injection, there were signs of AVN of the tibial plafond and the talus. There was also a nondisplaced fracture line in the talus that did not exist previously (Figure 3). There were no inciting traumatic events between the MRIs taken five and seven months after the initial fall. Multiple MRIs have been taken since the initial discovery of AVN. Figure 4 demonstrates an MRI from September 2022, which shows no progression of AVN or collapse.

Treatment

The patient presented to our clinic 13 months after the initial injury, non-weight bearing in a CAM boot. We placed her on Fosamax 70 mg once weekly, 5000 units of vitamin D daily, and introduced an Enovis combined magnetic field bone growth stimulator for 30 minutes daily, in an effort to prevent articular collapse. She was gradually made weight-bearing as tolerated, as symptoms allowed, and her pain decreased. However, her anterior ankle pain and swelling persisted. After a follow-up monthly for three months, she continued to express that the pain was constant and exacerbated by walking and improved by rest. It was felt that the original soft tissue impingement in the anterior lateral scar tissue and a thickened Bassett's ligament that resulted from the sprain were still symptomatic. A 1% lidocaine injection was utilized to see if the pain that the patient was experiencing was coming from a local source within the ankle, such as soft tissue impingement and synovitis, which frequently occur after an ankle sprain. When the injection was given, the patient's pain immediately resolved within five minutes. Therefore, it was reasoned that there was likely soft tissue impingement or synovitis leading to pain and swelling. Although AVN can also give pain, it was not felt that the lidocaine injected into the joint would have relieved that pain. Therefore, the option of surgery was presented. The plan included arthroscopic debridement of synovitis and scar tissue and possible core decompression of the tibia and talus. The patient, however, opted to proceed only with arthroscopic debridement.

**Figure 5:**
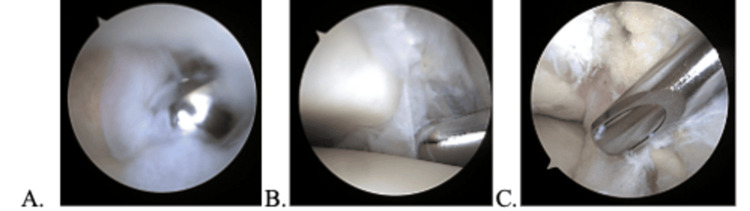
Debridement of a heavy scar on the anterior neck (A), resection of a thickened Bassett’s ligament (B), and resection of a meniscal band (C).

**Figure 6:**
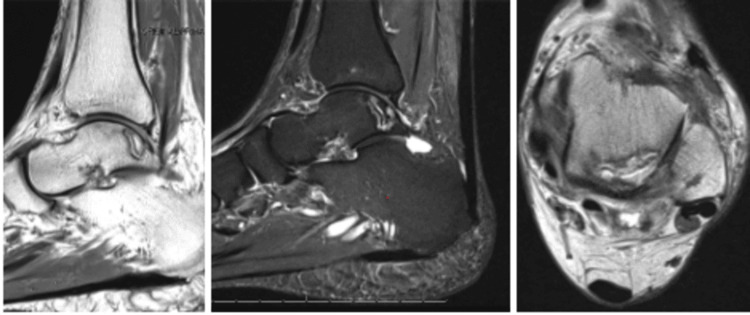
MRI from July 12, 2023, demonstrates resolution of tibial AVN, healing of the talus fracture with persistent AVN posterior lateral talar dome. AVN: avascular necrosis

Left ankle arthroscopic debridement was performed 19 months after the initial injury. During the procedure, extensive scar tissue was debrided (Figure 5). One year postoperatively, the patient reports ongoing ankle pain, although less than pre-operatively. She can ambulate in a regular shoe without bracing and has minor activity limitations. An MRI four months after surgery demonstrates resolving tibial AVN and stable talar AVN without collapse with a healed talar body fracture (Figure 6). The current plan is to continue to observe the patient.

## Discussion

The development of AVN in the talus and tibial plafond after corticosteroid injection has not been reported. Upon review of this patient’s history, no other obvious etiology can be identified. Although she utilized a Medrol dose pack, this was many months before the corticosteroid injections and the onset of worsening pain. AVN has been reported after injection into other major joints, although the exact mechanism of action is not known. Possibilities include corticosteroid-induced AVN or aggravation of already present AVN. There are many complex pathologic effects of corticosteroid administration, including an increase in intraosseous extravascular pressure leading to a decrease in blood flow and dysregulated bone formation [6].

AVN in the talus can be identified on radiography by an increase in the talar dome opacity and by articular collapse and fragmentation [7]. On MRI, AVN presents as a low signal on T1-weighted images and variable signal on T2-weighted images. The “double line” sign is considered pathognomonic but less common [8].

Management of AVN in the talus varies depending on the extent of the osseous involvement. If there is no collapse of the joint, core decompression and vascularized pedicle bone graft have been successful options for treating osteonecrosis [1]. If the joint has collapsed, ankle fusion, tibiotalar calcaneal fusion, or arthroplasty, including total talus replacement, have been shown to be successful [8]. In this patient’s case, we opted to observe the AVN and to only treat the soft tissue impingement syndrome. She has improved clinically but is still symptomatic and will likely have permanent activity restrictions. Her radiographs from March 2024, approximately two years after the development of the AVN, demonstrate no evidence of collapse or arthritis.

## Conclusions

Although exceptionally rare, AVN after an intra-articular corticosteroid injection must be considered as a cause of persistent ankle pain. Advanced imaging will be needed to diagnose and plan the treatment of the AVN.
